# Nonulosonic acids contribute to the pathogenicity of the oral bacterium *Tannerella forsythia*

**DOI:** 10.1098/rsfs.2018.0064

**Published:** 2019-02-15

**Authors:** Susanne Bloch, Markus B. Tomek, Valentin Friedrich, Paul Messner, Christina Schäffer

**Affiliations:** Department of NanoBiotechnology, NanoGlycobiology unit, Universität für Bodenkultur Wien, Muthgasse 11, 1190 Vienna, Austria

**Keywords:** cell surface display, periodontitis, polymicrobial community, protein *O*-glycosylation, sialic acid-like sugar

## Abstract

Periodontitis is a polymicrobial, biofilm-caused, inflammatory disease affecting the tooth-supporting tissues. It is not only the leading cause of tooth loss worldwide, but can also impact systemic health. The development of effective treatment strategies is hampered by the complicated disease pathogenesis which is best described by a polymicrobial synergy and dysbiosis model. This model classifies the Gram-negative anaerobe *Tannerella forsythia* as a periodontal pathogen, making it a prime candidate for interference with the disease. *Tannerella forsythia* employs a protein *O*-glycosylation system that enables high-density display of nonulosonic acids via the bacterium's two-dimensional crystalline cell surface layer. Nonulosonic acids are sialic acid-like sugars which are well known for their pivotal biological roles. This review summarizes the current knowledge of *T. forsythia'*s unique cell envelope with a focus on composition, biosynthesis and functional implications of the cell surface *O*-glycan. We have obtained evidence that glycobiology affects the bacterium's immunogenicity and capability to establish itself in the polymicrobial oral biofilm. Analysis of the genomes of different *T. forsythia* isolates revealed that complex protein *O*-glycosylation involving nonulosonic acids is a hallmark of pathogenic *T. forsythia* strains and, thus, constitutes a valuable target for the design of novel anti-infective strategies to combat periodontitis.

## 1. Introduction

### 1.1. Periodontitis

To proliferate and persist in the oral cavity, bacteria tend to live in biofilms which are clinically described as oral plaque. As highly complex and dynamic polymicrobial communities these biofilms provide protection from shear forces and host immune responses. In a healthy individual, oral bacteria exist in a natural balance with the host. Different factors such as smoking, diabetes, genetic predisposition or poor dental hygiene can cause the community to become dysbiotic, enabling potentially pathogenic bacteria to increase in numbers and cause persistent infections, such as periodontitis. Periodontitis is a chronic inflammation of the gingiva and tooth-supporting tissues, including periodontal ligament and alveolar bone [1,2]. Up to 90% of the worldwide population is affected by periodontal disease, with 10–15% of patients suffering from severe forms, in which, if left untreated, inflammation causes irreversible tooth loss [3]. The dysbiotic oral microbial communities can mediate inflammatory pathology also at distant sites; periodontitis is associated with an increased risk to develop rheumatoid arthritis [4,5], atherosclerosis, cardiovascular diseases [6,7] and cancer [8]. Effective and targeted concepts for the treatment of the disease are currently unavailable. This might be due to the fact that the pathogenesis of periodontitis is still not fully understood. It involves a complex interplay of bacterial, genetic and environmental factors causing growth promotion of dysbiotic, predominantly Gram-negative, anaerobic bacteria that facilitate the progression of inflammation [3,9,10]. A number of potential pathogens promoting the onset of the disease have been identified through high-throughput sequencing, and metagenomic, metatranscriptomic as well as mechanistic studies [11–13]. These include the pathogens *Tannerella forsythia*, *Porphyromonas gingivalis* and *Treponema denticola* constituting the so-called ‘red complex’, a group of bacteria clearly associated with periodontal disease and classified as highly virulent [3,9,10,14].

**Figure 1.**
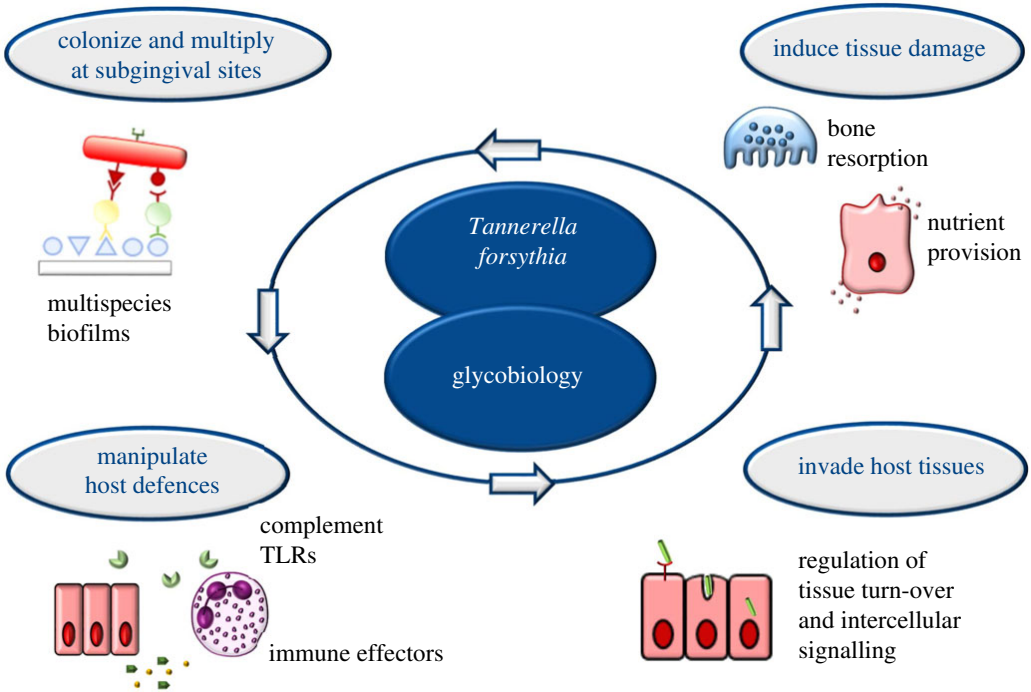
Glycobiology underpins the pathogenicity of the oral bacterium *Tannerella forsythia*. The unique protein-linked *O*-glycan of the bacterium was found to be involved in (i) the bacterium's establishment in oral plaque, (ii) manipulation of host defence mechanisms, (iii) adhesion to and invasion of host tissues and, eventually, (iv) tissue damage. (Online version in colour.)

The dysbiotic periodontal community is faced with a survival conundrum (figure 1): on the one hand, these bacteria need to evade immune-mediated killing; on the other hand, they require inflammation to procure nutrients from tissue breakdown such as degraded collagen peptides and haem-containing compounds [10]. Hence, immunosuppression, though a common evasion strategy of many pathogens, is not a viable option for inflammophilic bacteria. Periodontal bacteria can manipulate the interaction with host immune responses—such as neutrophils and complement—to enhance bacterial fitness. Major immune-subversive organisms probably use additional strategies to protect bystander bacteria and elevate the virulence of the entire microbial community, although most of these putative mechanisms have not been confirmed *in vivo*. For instance, the capacity of *P. gingivalis* to degrade and inactivate antimicrobial peptides might confer *in vivo* protection to bystander bacteria [15]. In the interaction with the gingival tissues and underlying immune cells, such as monocytes and macrophages which are present in high numbers in periodontal lesions, the ‘red complex’ bacteria induce the secretion of cytokines such as IL-1*β*, IL-6, IL-8 and TNF-α, which contribute to the exacerbation of inflammation [16–18].

Protein glycosylation is employed by numerous pathogenic bacteria to modulate the host immune response, and especially cell surface glycoconjugates such as glycosylated appendages like pili or flagella, play a vital role in orchestrating invasion and infection [19]. We and others have obtained evidence that a complex, protein-bound *O*-glycan which is displayed at high density on the bacterial cell surface [20] underpins the pathogenicity of *T. forsythia* [21–24].

### 1.2. The cell envelope of *Tannerella forsythia*

*Tannerella forsythia* was first isolated from advanced periodontal lesions in human oral cavities by Tanner *et al*. at the Forsyth Dental Center in Boston, MA, USA [25]. Originally designated *Bacteroides forsythus*, the bacterium was classified as an anaerobic Gram-negative member of the *Cytophaga–Bacteroides* family. When taxonomic analyses based on 16S rRNA sequences revealed that the species was more closely related to *Porphyromonas* than to *Bacteroides* it was moved to the family *Porphyromonadaceae* and formally reclassified to *Tannerella forsythia* [26,27].

*Tannerella forsythia* is a non-motile bacterium with filamentous cell morphology when grown with external supply of the essential cell wall sugar *N*-acetylmuramic acid (MurNAc) [28]. Physiologically, human-derived *T. forsythia* strains can be identified based on the following criteria: positive activity for (i) α-glucosidase, (ii) β-glucosidase, (iii) sialidase, (iv) trypsin-like enzyme, (v) negative indole production, (vi) requirement for MurNAc, (vii) colonial morphology and (viii) Gram-stain morphology from blood agar medium deficient in MurNAc [29].

Currently the genome sequences of seven *T. forsythia* strains are available in public databases; these are the ATCC 43037 type strain (accession number: NZ_JUET00000000.1), the isolates FCD 92A2 (NC_016610.1), KS16 (NZ_AP013045.1), 3313 (NZ_AP013044.1), UB4 (FMMN01000000.1), UB20 (FMMM01000000.1) and UB22 (FMML01000000.1). Additionally, a periodontal health-associated *Tannerella* species was described recently—HOT-286 (clone BU063; NZ_CP017038.1)—which is the closest phylogenetic relative of *T. forsythia* [30–32].

**Figure 2.**
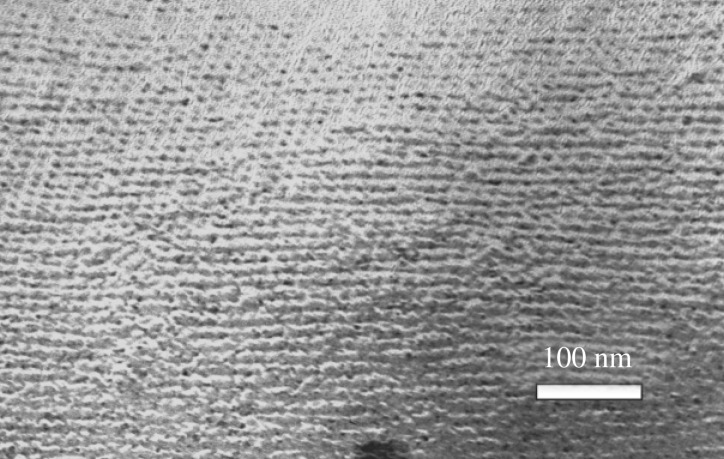
Square S-layer lattice on a *T. forsythia* ATCC 43037 cell as visualized by freeze-etching and metal-shadowing.

*Tannerella forsythia* shows a typical Gram-negative cell envelope consisting of a cytoplasmic membrane, a periplasm and an outer membrane that contains rough-type lipopolysaccharide [33]. In addition, the outer membrane of the bacterium is completely covered by a unique surface (S-) layer [34–36]. The S-layer represents a self-assembling, two-dimensional crystalline lattice formed of two different glycosylated proteins, TfsA and TfsB. The *T. forsythia* S-layer lattice has square symmetry with an overall lattice constant of 10.1 nm as revealed by a freeze-etched preparation of whole bacterial cells [35] (figure 2).

Compared to other S-layer-carrying bacteria, *T. forsythia'*s status is unique in two ways: (i) it is the only Gram-negative bacterium that is known to possess a glycosylated S-layer and (ii) its S-layer comprises two glycosylated proteins instead of just one [35,37]. Owing to their abundance and high molecular mass, TfsA (134.5 kDa) and TfsB (152.4 kDa) can be easily identified in SDS-polyacrylamide gels, where they migrate at apparent masses of roughly 230 and 270 kDa, respectively, owing to their glycosylation.

**Figure 3.**
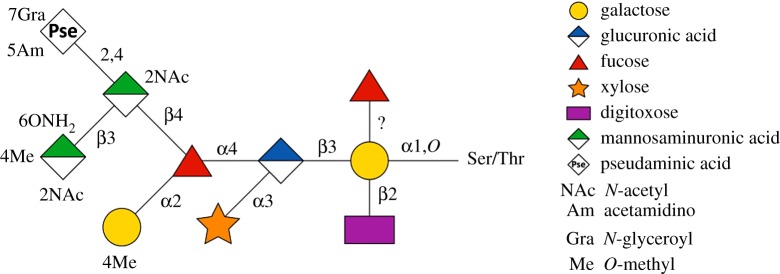
Structure of the unique *T. forsythia O*-glycan [21]. Monosaccharide symbols are shown according to the Symbol Nomenclature for Glycans [38]. (Online version in colour.)

The S-layer glycoproteins are the most abundant glycoproteins of *T. forsythia*. These and other outer membrane glycoproteins are equipped with a unique, complex *O*-linked decasaccharide [20,21]. The *T. forsythia O*-glycan (figure 3) is bound to distinct Ser and Thr residues within the three-amino acid motif D(S/T)(A/I/L/M/T/V) [20]. Notably, the glycan terminates strain-specifically with a modified nonulosonic acid, which can be either a modified pseudaminic acid as shown for the ATCC 43037-type strain, where an *N*-acetimidoyl (Am) and *N*-glyceroyl (Gra) substitution is present (Pse5Am7Gra), or a modified legionaminic acid, exemplified by strain UB4 [39]. Besides these sialic acid mimics, other unique S-layer glycan sugars present in the *T. forsythia O*-glycan are α-l-fucose (Fuc), digitoxose (Dig), xylose (Xyl), *N*-acetyl mannosaminuronic acid (ManNAcA) and *N*-acetyl mannosaminuronamide (ManNAcCONH_2_). The *T. forsythia O*-glycan, and bacterial cell surface glycans in general, represent the immediate contact zone of bacteria with the environment/host and are, thus, prone to act as specific ligands for cell–cell or cell–bacterium interactions, or to serve as virulence factors.

### 1.3. Nonulosonic acids

Nonulosonic acids (NulOs) are a class of 9-carbon sugars that are α-keto acids. The most abundant naturally occurring NulOs are the sialic acids (Sias) and their derivatives [40–42]. It is established knowledge that Sias play crucial roles in the life cycles of various organisms from eukaryotes to prokaryotes [43]. The various functions of Sias are to some extent opposed, generating competition in which hosts needs to maintain Sias for critical endogenous functions, while constantly changing them to avoid rapidly evolving pathogens that are either binding to or mimicking them. Available data are consistent with this evolutionary scenario [44]. Among the interaction partners of Sias are a family of Sia-binding molecules, mostly expressed by cells of the immune system, which have the potential to interact with sialylated glycans expressed on host cells or certain pathogens [20,45–47].

**Figure 4.**
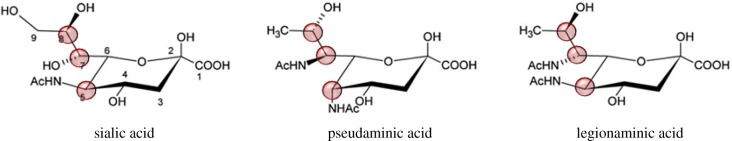
Structures of Sia and bacterial Sia-like sugars (NulOs) with the C5, C7 and C8 highlighted for modifications and stereochemical differences. (Online version in colour.)

While Sias are commonly present as terminal residues of many eukaryotic glycoconjugates and have also been identified in several prokaryotic polysaccharides, a number of NulOs appear to be unique to bacterial species [48–52]. Owing to their structural and biosynthetic similarities to Sias, these NulOs are also often referred to as Sia-like sugars (figure 4), the most studied of which are pseudaminic acids (such as 5,7-diacetamido-3,5,7,9-tetradeoxy-l-*glycero*-l-*manno*-NulO, Pse5,7Ac_2_) and legionaminic acids (such as 5,7-diacetamido-3,5,7,9-tetradeoxy-d-*glycero*-d-*galacto*-NulO, Leg5,7Ac_2_). For both pseudaminic acid (Pse) and legionaminic acid (Leg), several naturally occurring derivatives have been identified. These most commonly include substitutions of the *N*-acyl groups at the C-5 and C-7 positions, e.g. with *N*-acetyl or acetamido (Ac), *N*-acetimidoyl or acetamidino (Am), *N*-formyl (Fo) and *N*-hydroxybutyryl (Hb) groups [50].

Apart from some evidence for involvement in bacterial fitness and pathogenicity of distinct organisms, the function of Sia-like sugars as part of bacterial cell surface components is still largely unknown. It was suggested that bacterial NulOs could play a role as molecular mimics of eukaryotic Sia, thereby contributing to the pathogen's ability to evade or modulate the host's immune responses [45,53,54]. A recent study, for instance, proposes that Pse derivatives on the flagella of *Campylobacter jejuni* interact with host Siglec-10 to increase IL-10 expression, thus promoting an anti-inflammatory response and host persistence [55].

In contrast to the scarcity of evidence for their function, an increasing number of studies suggest that Pse and Leg derivatives are widespread among prokaryotes, where more than 20% of 1000 microbial genomes examined were found to encode a predicted NulO biosynthesis pathway [45]. Despite the similarities of Pse and Leg to Sias [48], the potential roles of these sugars in host–pathogen interactions are still poorly defined [56], and their distribution among microbes has not yet been systematically investigated.

## 2. Virulence factors of *Tannerella forsythia*

Through the expression of virulence factors, periodontal pathogens are able to colonize and persist in the host and promote the destruction of gingival tissues [57].

Several virulence factors that likely contribute to the pathogenicity of *T. forsythia* have been identified [58,59], and among them is the bacterium's S-layer [34,60,61]. S-layers are present as the outermost cell envelope layer of many bacteria and archaea and have a characteristic ‘lattice-like’ appearance with nanometre-scale periodicity [62]. They cover bacterial cells during all stages of the growth cycle in the form of a closed monolayer. S-layers provide to bacteria a protective coat with molecular sieve properties and generally function in the maintenance of bacterial integrity, display of bacterial components and interaction with the host non-immune and immune cells [63–65].

Other virulence factors of *T. forsythia* include the outer membrane glycoprotein BspA [66–68], envelope lipoproteins [69], KLIKK-proteases [70], trypsin-like [71] and PrtH [72] proteases, sialidases [73–76] and NanH [77], fucosidase [78], glycosidases [79], methylglyoxal [80] and lipopolysaccharide [33,81,82].

Recently, evidence was obtained that *T. forsythia* might use a very potent means of ‘biological warfare’ supporting its pathogenicity, namely outer membrane vesicles (OMVs) carrying virulent cargo [83]. OMVs are nanoscopic, spherical particles that are ubiquitously produced by Gram-negative bacteria and have been shown to contribute to the pathogenicity of many bacteria by enriching virulence factors and delivering them over long distances, superseding direct contact with the host [84,85]. *Tannerella forsythia* produces OMVs with a mean diameter of approximately 100 nm. In a shotgun proteomics approach, these were found to contain a number of known virulence factors and glycoproteins, supportive of a role of *T. forsythia'*s glycosylation in pathogenicity. Analysis of the expression and release of cytokines by macrophages and periodontal ligament cells challenged with *T. forsythia* OMVs showed a concentration-dependent inflammatory response that was significantly higher than that caused by whole cells, thus supporting the virulence potential of *T. forsythia* OMVs [83].

## 3. Nonulosonic acids and their potential association with periodontitis

### 3.1. Protein glycosylation in *Tannerella forsythia*

**Figure 5.**
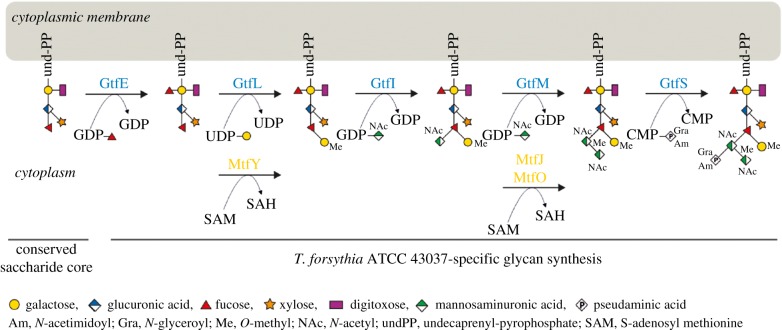
Biosynthesis pathway of the *T. forsythia* ATCC 43037 *O*-glycan. Modified after [21]. (Online version in colour.)

Protein *O*-glycosylation in *T. forsythia* is encoded in an approximate 27-kb, polycistronic glycosylation gene cluster working in concert with house-keeping functions of the bacterium [20,21]. Using a gene deletion approach targeted at predicted glycosyltransferases (Gtfs; named GtfSMILE) and methyltransferases (Mtfs; named MtfJOY) encoded in this gene cluster, in combination with mass spectrometry of the protein-released *O*-glycans, it was shown that the gene cluster encodes the species-specific part of the *T. forsythia* ATCC 43037 decasaccharide and that this is assembled on a pentasaccharide core from nucleotide-activated sugars in a step-wise fashion [21] (figure 5). The core was previously proposed to be conserved within the Bacteroidetes phylum, to which *T. forsythia* is affiliated [86], and its biosynthesis is encoded elsewhere on the bacterial genome. Upon synthesis of the pentasaccharide core on an undecaprenyl phosphate lipid carrier, the first carbohydrate residue of the species-specific glycan, a Fuc residue, is conferred by the glycosyltransferase GtfE to the reducing-end Gal of the glycan. The glycan is subsequently elongated with a Gal residue which is transferred by GtfL onto the core Fuc and methylated by MtfY. The assembly of the three sugar branch, consisting of a ManNAcA residue (transferred by GtfI), a ManNAcCONH_2_ residue (transferred by GtfM), which is methylated by either MtfJ or MtfO, and a Pse5Am7Gra residue (transferred by GtfS), completes the synthesis of the decasaccharide. A putative oligosaccharyltransferase (OTase) transferring the glycan onto the acceptor proteins could not be identified in the *T. forsythia* genome. This leaves the question open of whether this *O*-glycan is synthesized by an OTase-dependent or OTase-independent mechanism [87–90].

Since, in addition to the S-layer proteins TfsA and TfsB, also other proteins of *T. forsythia* are targeted by this protein *O*-glycosylation system [20], it is referred to as ‘general protein *O*-glycosylation system’. The species-wide conservation of the respective general protein *O*-glycosylation gene cluster was assessed by sequence comparison of the ATCC 43037 type strain with six other publicly available *T. forsythia* genomes (i.e. *T. forsythia* UB20, FDC 92A2, UB4, KS16, UB22, 3313). In all of the analysed *T. forsythia* genomes, general protein *O*-glycosylation gene clusters of comparable size, content and organization were identified [21]. Notably, these gene clusters start at the 5'-end with a Wzx-like flippase encoding gene for translocation of the glycan moiety to the periplasmic space prior to protein transfer, and terminate at the 3'-end with the *gtfE* gene encoding a fucosyltransferase initiating the biosynthesis of the *T. forsythia*-specific part of the *O*-linked decasaccharide. Further, two genes encoding enzyme proteins for the synthesis of UDP-linked ManNAcA (WecB, WecC) and one putatively involved in the Am-modification of the Pse residue were identified in the gene clusters. The major difference found between the analysed protein glycosylation gene clusters was in the alternate presence of six genes encoding the biosynthesis pathway for either CMP-Pse (in strains ATCC 43037 and UB20) or CMP-Leg (in strains FDC 92A2, UB4, KS16 and UB22), respectively.

The corresponding region in the genome of the periodontal health-associated *Tannerella* species isolate HOT-286 [31] showed a different gene composition lacking CMP-NulO pathway genes and most of the other genes commonly found in the pathogenic strains. Ongoing research on this difficult-to-grow *Tannerella* sp. [30] is assessing its glycosylation potential [32] as a basis to dissect a potential role in periodontal health and disease.

### 3.2. Biosynthesis and occurrence of nonulosonic acids in *Tannerella forsythia* isolates

For the incorporation of Pse or Leg at terminal position of an otherwise mostly identical *O*-glycan (except for one methyl group) [39], their CMP-activated forms are necessary. These are encoded by six pathway genes, each, named *pseBCHGIF* and *legBCHGIF*, respectively. The elucidation of the NulO biosynthetic pathways of *T. forsythia* relied primarily on knowledge of the Pse and Leg biosynthesis pathways in *Helicobacter pylori* [91] and *Campylobacter jejuni* [92], respectively. In a study by Fiedrich *et al*., CMP-NulO pathway enzymes from *T. forsythia* ATCC 43037 (CMP-Pse) and UB4 (CMP-Leg) were produced as recombinant proteins and functionally characterized [39]. In both cases, CMP-NulO is formed by the subsequent action of six enzymes starting from nucleotide-activated *N*-acetylglucosamine (GlcNAc). While the pathway to Pse uses UDP-GlcNAc as a precursor, Leg biosynthesis involves GDP-GlcNAc. Apart from using different sugar nucleotides, the biosynthetic routes for Pse and Leg differ in the reactions performed at the C-2, C-4 and C-5 positions of the hexose intermediates, which results in stereochemical differences at C-5, C-7 and C-8 positions in the final NulO [39]. In the first enzymatic step of the Leg pathway, the dehydratase LegB catalyses a C-4,6 dehydration, while the corresponding enzyme of the Pse pathway, PseB, additionally performs a 5-epimerization reaction. In the subsequent amino transfer at C-4 a stereochemical distinction as an axial (PseC) versus an equatorial (LegC) addition of the amino group is performed. After N-4 acetylation (PseH/LegH), NDP removal catalysed by LegG results in C-2 epimerization, whereas the corresponding enzyme of the Pse biosynthesis pathway, PseG, only possesses NDP-sugar hydrolase activity. In both NulO routes, the formation of the 9-carbon-backbone sugar is catalysed by homologous enzymes (PseI/LegI) via the condensation of a 6-carbon hexose intermediate with the 3-carbon molecule pyruvate. In the final enzymatic step, accomplished by PseF/LegF, CMP activates the free NulO residue using cytidine triphosphate. Considering that in *T. forsythia* strains unique NulO derivatives are found, i.e. Pse5Am7Gra in strain ATCC 43037 or modified Leg in strain UB4, but no modifying enzyme candidate could be reliably identified in the respective *T. forsythia* genomes, deviations from the outlined scenario would be anticipated to occur either within the NulO biosynthetic pathway or post CMP-NulO biosynthesis [39]. Since knowledge about the identity and function of NulO-modifying enzymes is scarce, the elucidation of the biochemical mechanism and genetic basis for the Am- and Gra-substitution observed in the ATCC 43037 type strain remains a major challenge.

In the so far analysed, limited number of *T. forsythia* genomes, the genetic potential for CMP-Leg biosynthesis seems to be more prevalent than that for CMP-Pse biosynthesis [21]. However, this finding needs to be validated by bioinformatic inspection of further genomes and accompanied with glycan analysis of the respective *T. forsythia* glycans. It should be noted that the genes of either CMP-NulO pathway have been found to be transcribed at a high level in different oral plaque samples containing *T. forsythia* (S. Bloch, S. Eick, C. Schäffer, unpublished data); this might underline the biological importance of NulOs in the physiology and/or pathogenicity of *T. forsythia*.

### 3.3. Protein *O*-glycosylation in *Tannerella forsythia* implicates stringent nonulosonic acid transferases

Based on the identification of CMP-NulO biosynthesis genes in all analysed genomes of pathogenic *T. forsythia* strains and the absence of those in the genome of the periodontal health-associated *Tannerella* isolate [21], we sought to characterize the NulO transferase from strains ATCC 43037 and UB4, the glycans of which terminate in a Pse5Am7Gra and modified Leg residue, respectively [39]. The enzyme would catalyse the transfer of either NulO on a proximal ManNAcA residue within the *O*-glycan structure (cf. figure 3).

Despite the predictably wide occurrence of NulOs in bacteria, no experimental evidence of a corresponding NulO transferase has been reported in the literature until research was carried out with the *T. forsythia* enzymes. The motility-associated factor Maf1 predicted to be involved in the transfer of Pse onto the flagellin of *Aeromonas caviae* was considered as a candidate Pse transferase [93,94]. With regard to Leg transferases, no predictions are presently available, neither in the literature nor in databases. Interestingly, selected sialyltransferases, i.e. porcine ST3Gal1, *Pasteurella multocida* sialyltransferase, *Photobacterium α*2,6-sialyltransferase and *Neisseria meningitidis* MC58 *α*2,3-sialyltransferase, were shown to accept CMP-Leg5Ac7Ac as a donor substrate to replace Sia as terminal sugar [95,96].

Bioinformatic analyses of the general protein *O*-glycosylation gene clusters provided the candidate genes *gtfS* (*Tanf_01245*; strain ATCC 43037) and *TFUB4_00887* (strain UB4), encoding a putative Pse and a Leg derivative transferase, respectively. The predicted transferases GtfS (445 amino acids; calculated molecular weight, 51.9 kDa) and TFUB4_00887 (442 amino acids; calculated molecular weight, 51.3 kDa) share an identical 241-amino acid long C-terminal domain and have an overall amino acid sequence identity of 81% [97]. A DXD motif starting at position D205 (ATCC 43037) and D202 (UB4), respectively, is present at the beginning of the conserved C-terminal domain. This short motif is found in many glycosyltransferase families, which add a range of different sugars to other sugars, phosphates and proteins. All DXD-containing glycosyltransferases use nucleoside diphosphate sugars as donors and require divalent cations [98]. However, DXD-motifs are usually absent in sialyltransferases [99], which do not require divalent metal ions for enzymatic activity. Instead, two recently identified functional motifs (D/E-D/E-G and HP), found in *Neisseria meningitidis* (NmB-polyST) and *Pasteurella multocida* (PmST1), are highly conserved in bacterial sialyltransferases and important to enzyme catalysis and CMP-Neu5Ac binding [100]; these are also present in the putative NulO transferases of the analysed *T. forsythia* strains. Thus, motif-wise the *T. forsythia* enzymes seem to be hybrids of glycosyltransferases and sialyltransferases.

Single-gene knock-out mutants targeted at either NulO transferase were analysed for S-layer *O*-glycan composition by ESI-MS, revealing the loss of the Pse5Am7Gra residue (ATCC 43037) and the Leg derivative (UB4), respectively, and thereby confirming the predicted activity of the enzymes in the glycan biosynthesis pathway. The substrate of either transferase is the fully modified CMP-NulO as was confirmed by the analysis of the cellular pool of nucleotide-activated sugars, where in *T. forsythia* ATCC 43037, CMP-Pse5Am7Gra with *m/z* = 683.2 and in *T. forsythia* UB4, modified CMP-Led with *m/z* = 654.3 were identified [39].

To learn about the substrate specificity of the *T. forsythia* Pse5Am7Gra and Leg derivative transferases cross-complementation experiments were performed. Complementation of *T. forsythia* ATCC 43037 *ΔgtfS* and *T. forsythia* UB4 *ΔTFUB4_00887* with the non-native enzyme could not restore the native *O*-glycan phenotype, despite identity of the underlying *O*-glycan structure. Thus, it is conceivable to assume that GtfS from *T. forsythia* ATCC 43037 and TFUB4_00887 from *T. forsythia* UB4 possess high stringency for the CMP-activated NulO substrate. Whether stringency relates to the stereoisomery or the modifications of the NulOs or both remains to be investigated.

## 4. Nonulosonic acids in the biofilm lifestyle of *Tannerella forsythia*

### 4.1. Monospecies biofilm

As part of the multispecies biofilms that constitute the dental plaque [3,9,10], the ability of *T. forsythia* to form biofilms is a vital prerequisite for the bacterium's survival in its natural environment. The presence of the *T. forsythia O*-glycan and its charged sugar residues (cf. figure 3) on the S-layer influences the physicochemical properties and hydrophobicity of the cellular surface and, thereby, has implications for the interaction of the bacterium with its environment. Nonulosonic acids in particular have long been implicated in facilitating bacterial virulence and survival within the host and impacting biofilm formation [101,102]. For *T. forsythia*, there has been obtained evidence that the glycosylated S-layer as an entity plays a role in the bacterium's biofilm lifestyle*.* In a proteomic analysis, the two S-layer proteins TfsA and TfsB were found to be upregulated in biofilms in comparison with planktonic cells [103], while in a different study, deletion of the *T. forsythia* S-layer led to an increase in monospecies biofilm formation attributing an inhibitory effect of the S-layer proteins on biofilm growth [104]. While these studies did not take into consideration a potential role of the individual sugars present in the attached *O*-glycan, first insight into the role of the *O*-glycan in this process was gained by studies on glycan mutants showing that a three-sugar truncation of the *O*-glycan including the Pse, ManNAcA and ManNAcCONH_2_ of *T. forsythia* ATTC 43037 (*T. forsythia ΔwecC*; UDP-*N*-acetyl-d-mannosaminuronic dehydrogenase deletion mutant) resulted in an increased biofilm formation when the bacteria were cultivated in an untreated polystyrene culture dish [105]. Friedrich *et al.*, on the other hand, reported on a promoting role of the terminal NulO derivatives (both Pse and Leg derivative in the different *T. forsythia* strains) in monospecies biofilm formation on a mucin-coated surface [39]. Not only the deletion of the terminal NulO, as well as the three-sugar truncation in *T. forsythia* ATTC 43037 *ΔwecC*, but also the complete deletion of the S-layer and attached glycan in *T. forsythia* ATTC 43037 *ΔtfsAB* resulted in a decreased capability of these mutants to form monospecies biofilms [106]. These contradictory results of monospecies biofilm experiments, with an increase in biofilm formation on untreated polystyrene plates upon glycan truncation [105] and a reduction of it on a mucin-coated plate [39,106], showed that biofilm behaviour is influenced by the properties of the surface provided for attachment. The coating with mucin renders the otherwise hydrophobic polystyrene surface highly hydrophilic [107], and influences how *T. forsythia* strains lacking one or more charged sugar residues can adhere to this surface. Through hydrophobic interactions, steric forces and charge effects, adhesion and bacterial interaction are influenced [108] and this effect becomes especially apparent when the bacterial cell surface composition is altered. The contribution of the cell surface, in particular the terminal Pse and Leg derivatives on interbacterial interaction was reflected in an enhanced autoaggregation of *T. forsythia* strains lacking the terminal NulO as well as the terminal three-sugar branch [106].

Functional analyses characterizing the growth and biofilm behaviour of *T. forsythia* strains differing in the display of either Pse5Am7Gra (ATCC 43037) or Leg derivative (UB4) on their surface as well as select cell surface mutants thereof, not only showed a contribution of the cell surface composition to the growth behaviour of *T. forsythia*, but also discovered differences in the growth characteristics of the two wild-type strains [106]. In a planktonic and monospecies biofilm setting as well as in a multispecies biofilm model, the two strains differed in their growth characteristics, with the clinical isolate *T. forsythia* UB4 generally growing to higher cell numbers than the *T. forsythia* ATCC 43037-type strain, suggesting strain-specific differences in the adaptation to varying environments [106].

### 4.2. Multispecies subgingival biofilm model

In the oral cavity, *T. forsythia* has to be able to survive in the environment of the multispecies biofilms. Using a subgingival biofilm model incorporating 10 different species of oral bacteria and thereby mimicking the native situation in the oral cavity [109], the extent to which *T. forsythia* uses its surface glycosylation to persist in its natural habitat was analysed [106].

As already observed in monospecies biofilm experiments, this study showed that the two *T. forsythia* strains—ATCC 43037 and UB4—differed considerably in their behaviour in the 10-species biofilms [106]. Comparison between the two strains showed that the latter was detected in much higher cell numbers in the multispecies consortium and both strains exhibited a different localization within the biofilm structure, as determined by confocal laser scanning microscopy (CLSM) [106].

**Figure 6.**
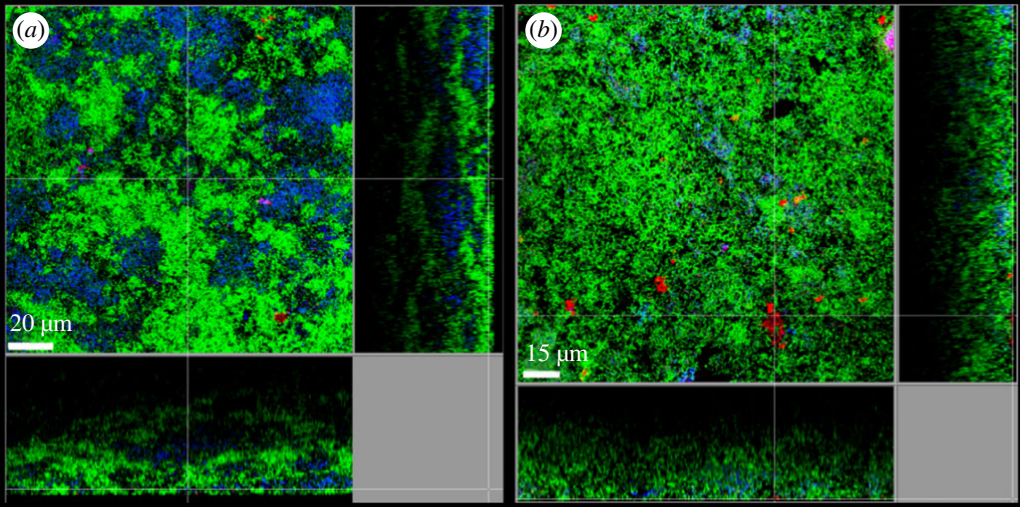
Fluorescence-*in-situ*-hybridization staining of fixed multispecies biofilms showing the localization of *T. forsythia* ATCC 43073 WT (*a*) and the NulO deficient mutant *T. forsythia* ATCC 43037 *ΔpseC* (*b*). Red: *T. forsythia*; cyan: *P. gingivalis* (*a*)/ *T. denticola* (*b*); green: non-hybridized cells (DNA staining YoPro-1 + Sytox). A representative area for one disc each is shown with a top view in the middle panel and side views with the biofilm–disc interface directed towards the top view. Scale bars, (*a*) 20 µm and (*b*) 15 µm. Both *T. forsythia* strains can be clearly detected in the form of microcolonies at the surface of the biofilm. Alteration of the surface glycosylation does not influence the bacteria's capability to grow in the multispecies consortium.

Contrary to expectations raised by findings of monospecies biofilm studies, truncation of the *O*-glycan or ablation of the S-layer as a whole did not influence the bacterium's capability to grow in the multispecies biofilms but modulated interbacterial interaction in the microcolonies within the biofilms, again highlighting the importance of the bacterial cell surface composition in this process [106]. The presence of the S-layer deficient *T. forsythia* ATCC 43037 *ΔtfsAB* in the multispecies biofilms led to an decrease in *Campylobacter rectus* cell numbers suggesting a growth impeding effect of the S-layer on this bacterium; however CLSM analysis and autoaggregation assays revealed this to be independent of a direct contact between the two species [106]. In CLSM, however, a strong co-localization of *T. forsythia* ATCC 43037 *ΔwecC* with *P. gingivalis* was noticeable [106]. Interestingly, a synergistic interaction between the two species had also been described using the same *in vitro* biofilm model showing reduced growth of *T. forsythia* in multispecies biofilms containing a *P. gingivalis* Lys-gingipain-deficient strain [110]. Outside of the multispecies consortium however, increased coaggregation between *P. gingivalis* and this mutant compared to the other *T. forsythia* strains analysed could not be identified [106]. These multispecies biofilm experiments suggest that the glycosylated S-layer is not vital for the establishment of the bacterium within the polymicrobial consortium but can influence the interaction with other species such as *C. rectus* and *P. gingivalis*. Contrary to a monospecies setting, the NulOs were not found to influence biofilm formation in the *in vitro* multispecies model [106] (figure 6).

Notably, decoupled from its cell surface composition, in the absence of early colonizing streptococci from the multispecies oral biofilm, the structural arrangement of *T. forsythia* within the biofilm is affected, changing from the formation of tight bacterial clusters within the biofilm to a more dispersed distribution [111].

## 5. *Tannerella forsythia* cell surface composition and immune response

### 5.1. Immune response to the *Tannerella forsythia* S-layer

The immunogenic potential of the *T. forsythia* S-layer was recognized in early studies investigating the IgG responses of human sera to purified TfsA and TfsB [112]. Their role as virulence factor was proposed following experiments showing that the S-layer proteins possess haemagglutination activity, mediate adherence and facilitate the invasion of epithelial KB cells [113].

By using *T. forsythia* ATCC 43037 wild-type cells in comparison to the S-layer-deficient *ΔtfsAB* mutant, Sakakibara *et al.* supported the evidence that the glycosylated S-layer of *T. forsythia* ATCC 43037 is involved in the adherence to and invasion of human epithelial cell-like gingival carcinoma cells (Ca9–22) and KB cells; this suggested that the *T. forsythia* S-layer plays an important role in the initiation stage of periodontal disease [60].

Another study comparing the *T. forsythia* ATCC 43037 wild-type with the *ΔtfsAB* mutant found that the S-layer conferred increased resistance to both calf and human non-heat-inactivated serum and inhibited deposition of complement factor C3b, a potent agent in the opsonization of pathogens, on the surface of the bacteria [114].

Further, the glycosylated S-layer of *T. forsythia* ATCC 43037 was shown to suppress the production of proinflammatory mediators IL-1β, TNF-α and IL-8 in U937 macrophages and human gingival fibroblasts [61], at least at the early stage of infection. These cytokines are released by macrophages during the early phase of host cell stimulation and are associated with the acute phase of host response [115]. Both IL-1β and TNF-α may directly stimulate bone resorption *in vitro* and *in vivo* [116] or stimulate production of prostaglandin E2 [117,118], which, in turn, is a potent stimulator of bone resorption [119]. IL-8 attracts neutrophils into the inflamed tissue, promoting the development of acute inflammation [120].

A recent study revealed the macrophage-inducible C-type lectin receptor (Mincle) as an important receptor for macrophage sensing of *T*. *forsythia* ATCC 43037 and modulation of the immune response to the bacterium in terms of induction of both pro- and anti-inflammatory cytokine secretion [121]. Mincle is an FcRγ-coupled pathogen recognition receptor that recognizes in a Ca^2+^-dependent manner proinflammatory stimuli from fungal and bacterial pathogens, with suggested mannose/fucose/*N-*acetylglucosamine/glucose specificity [122]. This might be indicative of the *T*. *forsythia* ATCC 43037 S-layer *O*-glycan with branching fucose residues serving as a ligand for the receptor. However, in-depth research is needed to support this assumption.

### 5.2. Immunogenicity of the *Tannerella forsythia O*-glycan

The presence of a sialic acid-like sugar residue as a terminal decoration of the *T. forsythia O*-glycan [123] suggests a role of the glycan in the pathogenicity of the bacterium, especially in the context of immune evasion and molecular mimicry.

As discussed above, the presence of the *T. forsythia* S-layer as an entity is a prerequisite for the bacterium to adhere to and invade gingival epithelial cells [60] and delays the immune response by macrophages and gingival fibroblasts [61]. A possible contribution to this process by specifically the S-layer *O*-glycosylation was first presented in a study using a *T. forsythia* ATCC 43037 *ΔwecC* mutant [124]. The terminal branch of the *O*-glycan was found to regulate dendritic cell effector function, suppress Th17 responses and neutrophil infiltration into the gingival tissues, thereby facilitating the persistence of the bacterium in the host [23,124]. The availability of deletion mutants of *T. forsythia* ATCC 43037 and *T. forsythia* UB4 lacking only the terminal NulO derivative further allowed a dissection of the function of Pse and Leg derivative in the interaction with the host [22].

In human oral keratinocytes (HOK) and human monocytes, both representing cells of the first line of defence against invading microorganisms, infection with the two *T. forsythia* wild-type strains displaying either a Pse or Leg derivative yields preferential immune responses. Infection with *T. forsythia* UB4 (Leg) dampens the IL-1β response in monocytes when compared to the *T. forsythia* ATCC 43037-type strain (Pse), but contrary to the latter strongly induces IL-7, which had not been observed in response to the species before; in HOK challenged with either wild-type, *T. forsythia* UB4 elicits a higher IL-8 release [22]. The cell surface composition of the bacterium seems to be especially relevant in the interaction with HOK where the ablation of the NulO, truncation of the *O*-glycan by three sugars and the deletion of the S-layer in *T. forsythia* ATCC 43037 significantly increase IL-8 release by HOK when compared with the parental wild-type strain [22]. These findings are in accordance with previous experiments attesting to the S-layer having a role in delaying the immune response during *T. forsythia* infection [61], however for the first time demonstrating a contribution of the terminal Pse derivative in this process [22]. The data suggest that the surface glycosylation of *T. forsythia* plays a prominent role in the initial phase of infection. By the suppression of proinflammatory immune responses on the one hand it might not only enable *T. forsythia* itself to persist in the host, but also other biofilm bacteria could benefit from it. Tomek *et al.* further analysed the role of the *T. forsythia O*-glycan at the immune interface using glycosyltransferase mutants of the *T. forsythia* ATCC 43037-type strain displaying truncated glycans on their surface [21]. While these mutants, lacking either the terminal Pse5Am7Gra residue, the terminal trisaccharide branch or the complete species-specific part of the glycan exposing a conserved pentasaccharide core with a terminal Fuc residue, did not affect dendritic cell maturation, the cell surface composition did have an effect on T-cell maturation [21]. Truncation of the glycan down to the pentasaccharide core resulted in an enhanced differentiation of Th17 cells [21] confirming the suppressive role of the *T. forsythia*-specific glycan portion and possibly the terminal Pse5Am7Gra residue in this process [23,124]. While the display of NulOs at the cell surface seems to be especially vital for the bacterium's interaction with the gingival tissues, the complete *O*-glycan might promote *T. forsythia'*s persistence within the host through dampening the initial response to bacterial infection and maintaining a favourable T-cell environment, thereby orchestrating *T. forsythia*'s virulence potential and pathogenicity.

## 6. Critical discussion

The periodontal pathogen *T. forsythia* synthesizes an elaborate *O*-linked decasaccharide as decoration of its abundant S-layer and other cell surface proteins. This glycan terminates strain-specifically in a modified NulO, which can either be a Pse or a Leg derivative. The *O*-glycan can be dissected into a *T. forsythia*-specific portion containing the NulO, and a core region which [21] is proposed to be conserved over the Bacteroidetes phylum [86], to which *T. forsythia* is affiliated. Considering the increasing documentation of the occurrence of NulOs in pathogenic bacteria [45,49], the functional proof the NulO derivative transferases in *T. forsythia* strains is an important and necessary step to fully understand the biosynthesis of NulO-containing glycan structures [97] and to exploit these enzymes for engineering of novel sialoglycoconjugates.

For NulOs, major roles have been attributed in biology and disease, including involvement in bacterial biofilm formation and motility. The structural similarity of Pse and Leg to eukaryotic Sias indicates molecular mimicry as a basic strategy these pathogens may employ to evade the host immune response [48,125]. However, it should be noted here that Pse and Leg have different stereochemistry, making Leg a potentially better mimic of host Sias than Pse. Interestingly, *T. forsythia* strain specifically varies the NulO type on an otherwise overall identical glycan. A comparable situation has so far only been described for *C. jejuni*, *Campylobacter concisus* [126], *Acinetobacter* spp. [127] and *Photobacterium profundum* [45]. The evolutionary purpose for such structural variation remains a matter of speculation. It is conceivable that different NulO types play distinct roles in the molecular mimicry of host tissues, thus enabling the pathogen to avoid immune detection, modulate the host immune response and enter into host cells to escape immune surveillance [128]. Whether for *T. forsythia* this correlates with the status of oral plaque or the stage of periodontal disease remains to be investigated.

It has been doubtlessly shown that *T. forsythia* employs its unique, NulO-containing cell surface to colonize its niche within the polymicrobial oral biofilm and to orchestrate the immune response of resident host tissue and the immune system [22,106]. Studies with human macrophages and gingival fibroblasts demonstrated that the S-layer attenuates the host immune response by evading recognition by the innate immune system, at least at the early stage of infection [61]. There are indications that specifically the S-layer *O*-glycan is crucial for the modulation of host immunity through Th17 suppression [124]. With regard to the modulation of dendritic cell effector functions, it was specifically shown that the *T. forsythia*-specific glycan portion suppresses and the pentasaccharide core activates a Th17 response. A very recent study suggests a role specifically of the modified Pse residue (Pse5Am7Gra) present at terminal position on the *T. forsythia* ATCC 43037 *O*-glycan in facilitating immune evasion by dampening the response of epithelial tissues to initial infection [22]. Changes in the S-layer and surface glycosylation of *T. forsythia* might actually contribute to the bacterium's virulence potential by promoting structural arrangements within the biofilm. Whether this contributes to the immune evasion of the biofilm-associated species needs to be tested in functional interaction assays with host cells [106].

From bioinformatic analysis of available genomes of seven pathogenic *T. forsythia* strains and one periodontal health-associated *Tannerella* isolate for the presence of genes encoding the biosynthesis of CMP-activated NulOs as required for the incorporation of NulOs in the S-layer *O*-glycan, it can be concluded that complex, NulO-containing protein *O*-glycosylation is a hallmark of pathogenic *T. forsythia* strains; thus, we propose it as a valuable target for the design of novel antimicrobials against periodontitis.

## Data accessibility

This article has no additional data.
